# Multiplexed smFRET Nucleic Acid Sensing Using DNA Nanotweezers

**DOI:** 10.3390/bios13010119

**Published:** 2023-01-10

**Authors:** Anisa Kaur, Roaa Mahmoud, Anoja Megalathan, Sydney Pettit, Soma Dhakal

**Affiliations:** Department of Chemistry, Virginia Commonwealth University, Richmond, VA 23284, USA

**Keywords:** multiplex sensing, nanotweezers, single-molecule FRET, biosensors, miRNA biomarkers

## Abstract

The multiplexed detection of disease biomarkers is part of an ongoing effort toward improving the quality of diagnostic testing, reducing the cost of analysis, and accelerating the treatment processes. Although significant efforts have been made to develop more sensitive and rapid multiplexed screening methods, such as microarrays and electrochemical sensors, their limitations include their intricate sensing designs and semi-quantitative detection capabilities. Alternatively, fluorescence resonance energy transfer (FRET)-based single-molecule counting offers great potential for both the sensitive and quantitative detection of various biomarkers. However, current FRET-based multiplexed sensing typically requires the use of multiple excitation sources and/or FRET pairs, which complicates labeling schemes and the post-analysis of data. We present a nanotweezer (NT)-based sensing strategy that employs a single FRET pair and is capable of detecting multiple targets. Using DNA mimics of miRNA biomarkers specific to triple-negative breast cancer (TNBC), we demonstrated that the developed sensors are sensitive down to the low picomolar range (≤10 pM) and can discriminate between targets with a single-base mismatch. These simple hybridization-based sensors hold great promise for the sensitive detection of a wider spectrum of nucleic acid biomarkers.

## 1. Introduction

Sensors enabling multiplex detection of biomarkers are of great interest as they provide information on diseases while saving time, expenses, and resources [1,2,3]. More importantly, this strategy allows the detection of multiple analytes in one sample, and thus the efficacy and accuracy of diagnosis can be greatly enhanced [4,5,6]. This is because the detection of the aberrant expression of a single biomarker can be indicative of many different disease states resulting in false positives/negatives, thus reducing the overall reliability of patient disease assessments [3,7]. Although several fluorescence-based techniques such as microarrays and quantitative polymerase chain reactions (q-PCR) offer high-throughput, simultaneous detection of biomarkers, they come with their own limitations. For example, microarrays are semiquantitative, often require significantly complicated sample preparation steps, and suffer from low target specificity [8,9,10]. On the other hand, q-PCR methods allow target-specific and low-cost multiplex detection; however, they suffer from significant false-positive rates due to the challenges associated with primer design, leading to amplification of the false target [9,11,12]. Other multiplexing methods, such as surface-enhanced Raman spectroscopy (SERS), electrochemical biosensors, and quantum dots, do not require the target to be labeled or amplified but are limited in the number of reporter labels that can be used to produce well-resolved and nonoverlapping signals/currents [13,14,15,16,17].

In recent years, the use of single-molecule techniques, such as single-molecule FRET (smFRET), has shown great potential in the field of multiplexed sensing owing to their potential for sensitive and low-background detection of nucleic acid biomarkers [18,19,20,21]. However, many of these FRET approaches are based on the use of multiple excitation sources and complex labeling schemes, which further complicates the experiments, the analysis of data, and the interpretation of results [18,22,23,24,25]. In an effort to develop a multiplexed sensor that employs only one FRET pair by utilizing tunable inter-dye distances, we have recently demonstrated a novel sensor design called the interconvertible hairpin-based sensor (iHabS) [6]. However, because the detection signals relied heavily on the toe-hold-mediated strand displacement (TMSD) of the probe by the target, this two-step indirect sensing approach resulted in a moderate sensitivity (LOD ~200 pM) [6]. In fact, the pM level sensitivity is not strong enough to detect nucleic acid biomarkers in biological samples as the concentration falls in the low pM to fM range [10,26,27].

In order to improve upon this previous sensing strategy, we decided to focus on a realistic set of sequences in the detection of a known biomarker signature for triple-negative breast cancer (TNBC). The relevance of using TNBC biomarkers comes from the fact that it is a subtype of breast cancer that lacks the three common breast cancer receptors: estrogen, progesterone, and HER2, making it harder to detect [28,29]. Recent studies have shown that the deregulation of multiple miRNAs specific to a particular disease can be used as a signature for early-stage detection, which is of particular interest in the case of highly aggressive cancers such as TNBC [4,28,29]. Furthermore, previous studies have shown that the detection of two or more miRNAs known to be associated with TNBC provided a robust risk assessment of early breast cancer recurrence compared to the detection of only one miRNA [3,4,5,6,28]. In this regard, multiplexed detection of an miRNA signature specific to TNBC can be a valuable asset for early-stage diagnosis of this deadly disease.

Here, we present a DNA-based nanotweezer (NT)-like sensor for the sensitive, specific, and low-background detection of four specific sequences that are the mimics of TNBC miRNAs (miR-92a-1, NR_029508; miR-let7a-1, NR_029476; miR-652, NR_030381; and miR-107, NR_029524) [4,29,30,31]. The NR values represent the corresponding miRNA accession numbers found in the human genome nomenclature (HGNC). The key advantage of the nanotweezer design is that it avoids the slower TMSD process through direct binding. This is important because the direct hybridization sensing strategy allows sensitive detection by pushing the detection limit to a more biologically relevant concentration range of miRNAs (low pM or fM) [26,27]. By fine-tuning the imaging strategy and employing distance-tunable multiplexing with a single-molecule counting approach, we showed that each sensor has its own FRET signature and therefore this strategy can enable four-fold multiplexing. The four sensors presented here allow the detection of their respective targets with ≤10 pM detection limits and a background of nearly zero in the absence of the target. We also show that the sensors have a superior specificity to fully complementary targets compared to single-nucleotide mismatched targets. Altogether, the sensing approach that we developed shows a great potential in the fields of biotechnology, diagnostics, and biomarker analysis.

## 2. Materials and Methods

### 2.1. Materials

Tris(hydroxymethyl)-aminomethane (Tris), acetic acid, potassium chloride, glycerol, magnesium chloride hexahydrate, ethylenediaminetetraacetic acid disodium salt (EDTA), 6-hydroxy-2,5,7,8-tetramethylchroman-2-carboxylic acid (Trolox), and protocatechuate 3,4-dioxygenase (PCD) were all purchased from Fisher Scientific. PCD was suspended in a PCD stock buffer (pH 8.0), which contained 100 mM Tris−HCl, 50 mM KCl, 1 mM EDTA, and 50% glycerol, sterile-filtered, and stored at −20 °C. Biotinylated bovine serum albumin (bBSA) was purchased from Thermo Scientific. Protocatechuic acid (PCA) and streptavidin were purchased from VWR International, LLC. All DNA oligonucleotides were purchased from Integrated DNA Technologies (IDT) and primary stock solutions were prepared at 100 μM in filtered sterile water and stored at −20 °C until needed.

### 2.2. DNA Construct Assembly

In order to form the DNA constructs, constituent single-stranded DNA (ssDNA) oligonucleotides (Table S1) were thermally annealed at 1 µM concentrations in 1× TAE-Mg buffer (40 mM Tris, 20 mM acetic acid, 1 mM EDTA, 12.5 mM MgCl_2_, pH 7.4). The thermal annealing was carried out by slowly decreasing the temperature of the solution from 95 °C to 4 °C over a period of ~2 h in a thermal cycler as described in our previous publications [6,32].

### 2.3. Surface-Functionalization of Flow Cells

Flow cells were prepared as described elsewhere [32]. For single-molecule experiments, flow cells were functionalized by first incubating them with 1 mg/mL biotinylated BSA for 5 min, followed by incubating them with 0.2 mg/mL streptavidin for 2 min, before injection of the sample. Then, ~300 µL of 1× TAE buffer was flushed through the flow cell to remove unbound molecules before recording single-molecule videos.

### 2.4. Sample Preparation and Imaging

Fluorescence imaging was carried out as described previously, with some modifications [6,32,33]. Briefly, sensors (~20 pM) prepared in 1× TAE and an oxygen scavenging system, OSS (4 mM Trolox, 10 mM PCA, 100 nM PCD), were immobilized onto a functionalized quartz slide via biotin/streptavidin interaction, incubated for 30 s, and washed with 1× TAE-Mg buffer (40 mM Tris, 20 mM acetic acid, 1 mM EDTA, 12.5 mM Mg^2+,^ pH 7.4) containing OSS to wash away unbound assemblies. The target was prepared at the desired concentration in 0.02% BSA and 1× TAE containing 100 mM Mg^2+^ and OSS, injected onto the slide in 3 intervals with 1 min incubations between each interval, and incubated for 15 min, before washing with a no-salt buffer containing 1× TAE buffer and OSS to remove unbound target molecules. Single-molecule videos were acquired 5 min after injection of the wash buffer. Control experiments were performed similarly in the absence of a target. Moreover, in the multiplexing experiments, the molar ratio of the NT sensors was maintained at a 1:1:1:1 equimolar concentration, where the total concentration of the sensors was 20 pM (5 pM each). To obtain an image of the microscope slide, the Cy3 fluorophore was continuously excited using a green laser of 532 nm. An iXon Ultra EMCCD camera (512 × 256 pixels) was used to simultaneously record fluorescence emissions from Cy3 and Cy5 fluorophores through green and red channels, respectively, at a 100 ms time-resolution. Toward the end of each recorded movie, the red laser (λ = 639 nm) was turned on to excite Cy5 fluorophores and confirm the presence of an active FRET pair. All experiments were performed at room temperature (23 °C).

### 2.5. Data Acquisition and Analysis

Data acquisition was performed as outlined in previous publications [6,33]. Briefly, Single.exe software was used for data acquisition, and post-processing of data was performed using IDL and MATLAB scripts as described previously [33,34]. Those molecules that provided evidence of both single-Cy3 and single-Cy5 fluorophores with single-photobleaching events were manually selected for subsequent analysis. Intensity-time and FRET-time traces were constructed using Origin by selecting the first 300 frames of the data from the chosen single molecules, where the FRET efficiency (E_FRET_) was calculated using the equation *I*_A_/(*I*_D_ + *I*_A_); *I*_A_ and *I*_D_ represented the background-corrected fluorescence intensities of the acceptor and donor fluorophores, respectively [35,36]. A single molecule counting approach was employed to assess the percentage of anti-correlated molecules (i.e., molecules showing a decrease in donor intensity that is accompanied by an increase in acceptor intensity and vice versa) from all single molecules chosen. Of all molecules chosen, only those molecules which exhibited a clear anti-correlation between their donor and acceptor fluorescence intensities were considered to be positive signals indicating target binding. The number of molecules exhibiting anti-correlation over the total number of single molecules was reported as percentage. Standard deviations and averages for single-molecule experiments were acquired by randomly assigning molecules to three groups and analyzing and evaluating the percentage of anti-correlated molecules for each group.

## 3. Results and Discussion

### 3.1. Sensor Design and Working Principles

**Figure 1:**
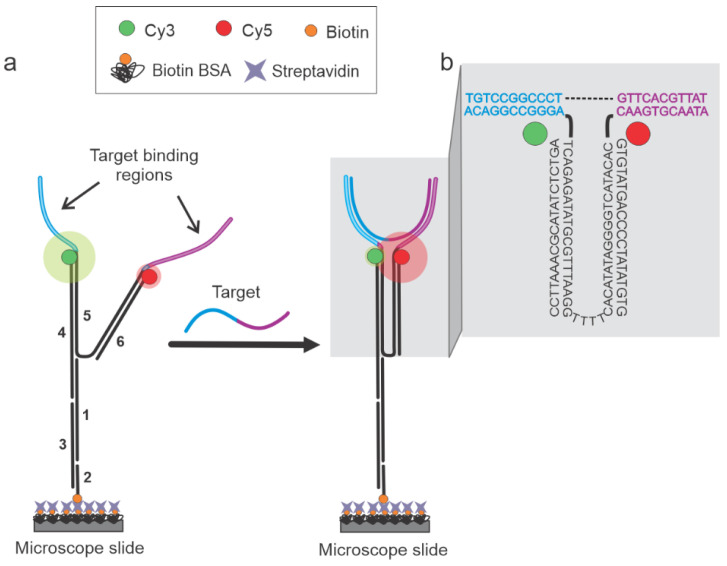
Working principle of the DNA nanotweezer (NT) sensor. (**a**) The sensor is made up of six strands (1: Strand 1, 2: Bio 5′ Comp, 3: Splint, 4: Cy3 Terminal, 5: NT-92a or NT-0, and 6: Cy5-Trunc, as identified in the Supplementary Table S1) and remains in the open conformation (low E_FRET_) in the absence of a target. Target hybridization switches the sensor to the closed conformation (high E_FRET_) since the target is complementary to one-half of each arm (target binding regions in blue and magenta). (**b**) Zoomed-in view of the NT, depicting the sequences for the NT-0 sensor specific for the DNA92a target (see Table S1 for sequences). The dashed line is used only to connect the two halves of the target sequence and no such gap exists in the real sequence.

In this study, a DNA-based NT sensor design was utilized for the sensitive, specific, and direct detection of short nucleic acid sequences to overcome the need for the indirect TMSD process. NT-based designs have been previously reported for their switch-like opening and closing capabilities upon target hybridization [37,38,39,40,41,42,43,44,45,46,47]. We decided to use these capabilities of NTs for clear on/off multiplexed sensing. The basic sensor design and working principles are outlined in Figure 1. Sensors were prepared via the thermal annealing of six constituent strands in 1× TAE-Mg buffer (see Section 2 and Table S1). The target-binding regions of the sensor (11 nucleotides each, Figure 1) were rationally designed to be complementary to the target sequence, where each region complemented one-half of a known target (~20–23 nucleotides in length). The rest of the strands were custom-designed and then incorporated accordingly to create the two fluorophore-labeled arms. One arm was extended to allow the incorporation of a biotin-labeled oligonucleotide for the immobilization of the sensor onto a streptavidin-functionalized slide surface. Each arm of the sensor contained either a donor (Cy3) or an acceptor (Cy5) fluorophore. The design is such that the two arms of the sensor can only come into proximity (closed conformation) in the presence of a fully complementary target sequence, producing a high FRET efficiency. In the absence of a target, the sensor exhibits a low-FRET open conformation. This sensor design was then multiplexed using the distance-tuning approach while still using the single FRET pair, as introduced in our previous publication [6]. By tuning the Cy3 and Cy5 distances in the closed conformation with the use of terminal and internal Cy3-labeled oligonucleotides, we intended to produce distance-dependent high-FRET efficiency values. The Cy3 oligonucleotides used had Cy3 labels 0, 5, 8, or 12 nucleotides (nt) away from the terminal 3′ end and therefore these sensors were named NT-0, NT-5, NT-8, and NT-12, respectively, and each of them was expected to produce distinguishable FRET states from one another.

### 3.2. Single-Molecule Characterization of Sensors

First, we validated the performance of the NT sensors individually via smFRET analysis using a total internal reflection fluorescence microscope [33]. Briefly, the fully annealed NT sensors were immobilized onto a quartz slide functionalized with biotinylated BSA and streptavidin via the biotin-labeled oligonucleotide incorporated into the sensor design. Unbound sensor molecules were washed away with an imaging buffer (1× TAE-Mg, pH 7.4) containing an oxygen scavenging system (OSS) to retard the photobleaching and photoblinking of fluorophores [48,49].

The target (100 pM) was injected and incubated on the microscope slide for 15 min in an imaging buffer containing 0.02% BSA and 100 mM Mg^2+^ to prevent non-specific binding of the nucleic acids and facilitate hybridization to the surface-immobilized sensors, respectively. Although lower concentrations of Mg^2+^ were also tested (2, 10, 25, and 50 mM Mg^2+^), less or no evidence of target binding was observed. Unfortunately, direct imaging in the presence of the higher concentrations of salt provided very noisy fluorescence intensity-time traces, with traces showing rapid unexplainable fluctuations without clear anti-correlation between the donor and acceptor, making it difficult to identify real single molecules. For this reason, we tried removing the Mg^2+^ after the target-binding step by adding a wash buffer (1× TAE buffer containing only OSS) before beginning the data acquisition process. Surprisingly, the target remained bound after the addition of the wash buffer, as indicated by the presence of molecules exhibiting a high FRET efficiency.

**Figure 2:**
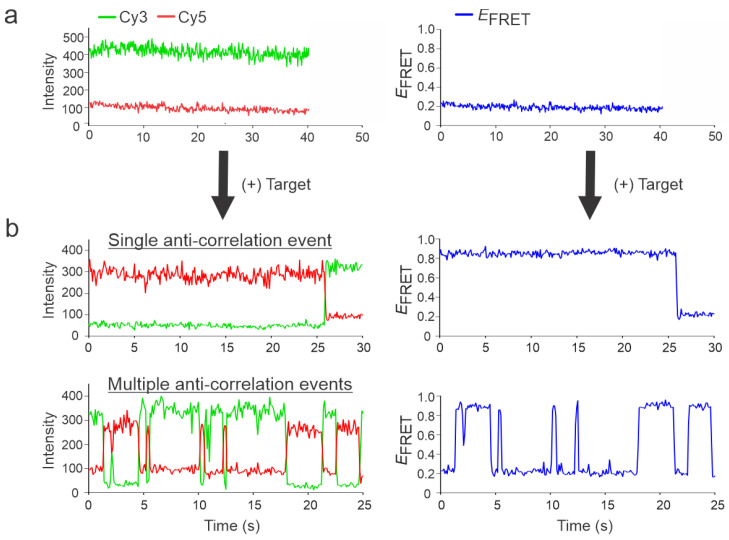
Representative intensity-time and E_FRET_-time traces in the presence and absence of the target. (**a**) No anti-correlation between the donor and acceptor fluorescence was observed in the absence of the target with a low FRET efficiency of ~0.2. (**b**) Addition of the target resulted in two types of traces: single anti-correlation events and multiple anticorrelation events, referred to as static or dynamic traces, respectively, along with an upward shift in the FRET efficiency of ~0.95. The data presented here belongs to the NT-0 sensor and its specific target, DNA92a.

**Figure 3:**
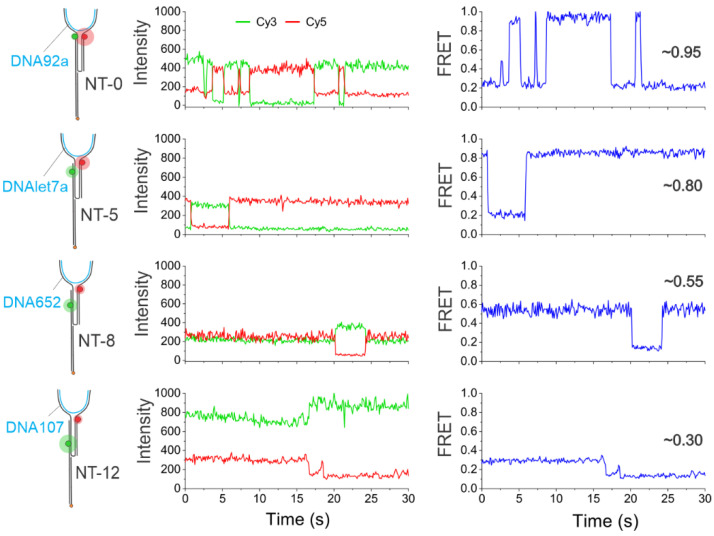
Representative intensity-time and E_FRET_-time traces showing either static or dynamic behaviors for each sensor (schematics shown on the left). The red and green spheres represent Cy5 and Cy3 fluorophores respectively and the blue lines represent the DNA targets. Distinctly different FRET efficiency values were observed for each NT sensor immobilized individually on the microscope slide. Specifically, NT-0, NT-5, NT-8, and NT-12 showed FRET efficiency values of ~0.95, ~0.8, ~0.55, and ~0.30, respectively. The target was added at 100 pM in all experiments. Each sensor was characterized individually.

Intensity- and FRET efficiency-time traces in the absence and presence of the target (DNA92a, which is a DNA mimic of miR-92a; see Table S1 for sequence details) using the NT-0 sensor were first analyzed (Figure 2). The (-) target control experiments were performed similarly; sensors were incubated with imaging buffer containing 100 mM Mg^2+^ for 15 min and washed with the wash buffer before imaging. As expected, in the absence of a target, the sensor molecules remained in the open conformation, exhibiting only a relatively static low-FRET efficiency of ~0.2 with no evidence of a high-FRET state, suggesting a background of zero for detection (Figure 2a). The static low-FRET efficiency of these swinging arms of the sensor could be attributed to the time-averaged FRET efficiency of many conformational states. However, after the introduction of a target, the single-molecule traces showed evidence of a high-FRET state of ~0.95 (Figure 2b). Interestingly, the traces which exhibited a high FRET state could be either static or dynamic. Here, a static trace refers to a single anti-correlation event between the donor and acceptor intensity-time traces, and a dynamic trace refers to multiple anti-correlation events. The majority of dynamic traces became very advantageous as they enhanced the reliability of the detection since such distinct behavior is easily visually recognizable. The dynamic traces observed for the NT-0 sensor fluctuated from low (~0.2) to high (~0.95) FRET states, suggesting an opening and closing of the sensor or a rapid binding/unbinding of the target from the binding regions on opposing arms (Figure 2b). We hypothesize that this binding/unbinding behavior is due to the lack of Mg^2+^ in the solution after addition of the wash buffer that serves to stabilize the hybridization. All other sensors (NT-5, NT-8, and NT-12) showed a similar binding/unbinding behavior with the common low-FRET state of ~0.2 but with distance-dependent high FRET states of ~0.80, ~0.55, and ~0.30, respectively, when their respective targets were introduced (Figure 3). In addition to the static (i.e., single anticorrelation) and the slow dynamic behaviors exhibited by the sensors (Figure 3), they also showed distinctly fast dynamics in the presence of the target. Typical traces showing the fast dynamics for individual sensors are shown in Figure S1.

### 3.3. Determination of Analytical Sensitivity and Specificity of NT Sensors

**Figure 4:**
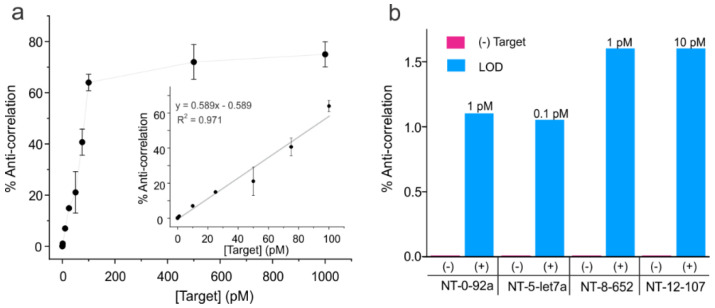
Analytical sensitivity of the NT sensors. (**a**) Full calibration curve showing the percentages of molecules showing anti-correlation in the donor-acceptor intensities out of the total single molecules against the target (DNA92a) concentration for NT-0. The inset depicts the linear region of the curve with a linear fit. Error bars represent standard deviations in the mean percentages of anti-correlated molecules obtained by assigning the molecules of a given target concentration into three groups (n = 3). A linear range up to 100 pM was observed, after which the data began to plateau, suggesting saturation of the NT-0 sensor. All data points were obtained by analyzing ~113–261 molecules. (**b**) Bar graph showing percentages of anti-correlation values for (-) target controls and the lowest target concentration tested to provide a positive signal (+ target data) for each target-specific NT sensor (i.e., limit of detection or LOD). All data were obtained from the analysis of ~125–238 molecules. The targets 92a, let7a, 652, and 107 refer to the DNA mimics as shown in Table S1.

After verifying that the sensors were able to detect a target by showing a distinct change in FRET efficiency compared to the sensors without a target, we determined the sensitivity of our sensors using a single-molecule-counting approach. Starting with the NT-0 sensor, we acquired single-molecule traces at different concentrations of the target (Figure 4a). The response as a function of target concentration was calculated as the number of anti-correlated molecules divided by the total number of single molecules in a dataset and then expressed as a percentage (see Data Analysis for details). We found that the limit of detection (LOD) was 1 pM for NT-0 and we were unable to obtain consistent results for anti-correlated molecules below this concentration. In addition, as shown in Figure 4a, linearity was seen up to 100 pM of the target (correlation coefficient (R^2^) = 0.971), after which the calibration curve plateaus suggested saturation of target binding, establishing the dynamic range of the sensor at ~100-fold (1 to 100 pM). The detection limits of NT-5, NT-8, and NT-12 and the background in the absence of the target were also evaluated using three other targets (DNA let7a, DNA652, and DNA107, respectively). Figure 4b shows that all sensors exhibited a background of ~zero, with no anti-correlated molecules observed in the absence of the target, and they were able to detect their respective targets down to low pM concentrations ranging from 0.1 pM to 10 pM depending on the target.

Taking this one step further, the specificity of the sensors was also evaluated by comparing the response of a fully complementary target to that of single-base-mismatched sequences. We chose NT-let7a as the model sensor to assess the aspect of specificity since it demonstrated the lowest detection limit among all four sensors (LOD = 0.1 pM). Mutant sequences were custom-designed to introduce mismatches at the center, right arm, or left arm of the target let-7a (Table S2). The rationale behind the use of these specific mutations was to incorporate mismatches at all possible sites that could lead to a loss of stable hybridization with the NT probe sequence, keeping the sensor in the open (low-FRET) conformation. The experimental protocol and conditions were similar to those applied for assessing the sensitivity of the sensors (see Section 2). Briefly, a near-saturating concentration of the mutant sequences (100 pM) was used and injected onto the sensor-immobilized slides in three intervals, each 1 min apart, and incubated for a total of 15 min, before washing with the no-salt buffer. Figure S2 displays some representative intensity-time traces and FRET traces for mutants 1–4. The vast majority of the NT-5 molecules showed around 0.2 FRET, indicating that the sensor was in the open unbound state. In further analyses of the traces, we found that mutants 1–3 displayed a false positive signal of ≤1%; however, mutant 4 showed a percentage anticorrelated signal of ~10% (Figure S3). The relatively high background signal for mutant 4 indicated the binding of this mismatched target via the unmutated half of the sequence that was G-rich. This postulation is well supported by the fact that mutant 3, with a mutation in the G-rich arm, showed a substantially lower signal (~1%). Nonetheless, the response of the sensor to the fully complementary target was much higher, ~80%, compared to the mutants (Figure S3), demonstrating the high specificity of the NT sensor.

### 3.4. Validation of the Multiplexed Sensing Method

**Figure 5:**
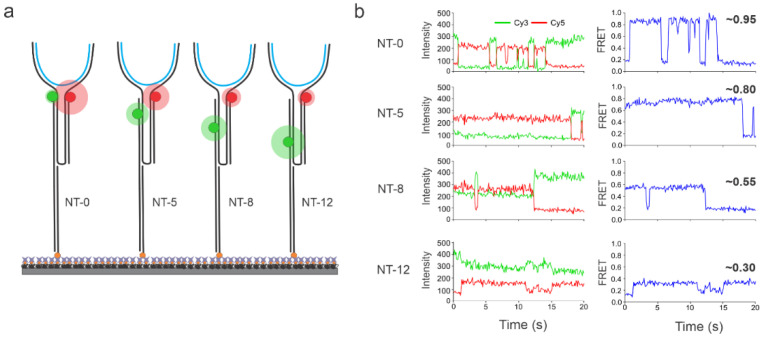
(**a**) Schematic representation of the proof-of-concept multiplexing experiment. The four NT sensors with different inter-dye distances used here were all designed to detect DNA-92a. The red and green spheres represent Cy5 and Cy3 fluorophores respectively and the blue lines represent DNA-92a. (**b**) Typical intensity-time traces from the multiplexed data with varying E_FRET_ values. The NT sensors were post-assigned after analyzing each trace’s high-FRET state and comparing it with the observed high-FRET states of each individual NT sensor.

To validate the multiplexing capability of the NT sensor, a mixture of the four sensors with different inter-dye distances was used in a model experiment using the very first NT-92a sensor, where all shared the same probe sequence and performed detections for the same target (DNA92a; see Table S1 for sequence details). The sensors were pre-mixed at an equimolar ratio and surface-immobilized onto a functionalized quartz slide (Figure 5a), imaged in the absence of the target, and re-imaged after incubation with 100 pM of a target for 15 min and washing with a wash buffer. Figure 5b shows the four different intensity-time traces and their corresponding FRET-time traces, as observed in the multiplexing experiments. Four different high-FRET states of around 0.95, 0.8, 0.55, and 0.30 were observed, which corresponded well with the experimental high-FRET state observed for the individual sensors. These results suggested that regardless of the position of the sensors immobilized on the slide surface, positive signals could be distinguished from the single-molecule traces—thereby allowing single-molecule counting.

## 4. Conclusions

To expand the application of single-molecule multiplexing in detecting nucleic acid biomarkers that are present at ultra-low concentrations (picomolar to femtomolar), such as those found in biological samples, we developed a DNA NT sensor that offers direct binding of the target to the sensor. Multiple sensors with slightly different donor–acceptor distances and the direct binding of targets allowed the detection of four different DNA mimics of miRNAs with a detection limit ranging from 0.1 pM to 10 pM. In addition, low pM detection of four targets was made possible without requiring target labeling and amplification. Due to the unique ability of these sensors to exhibit dynamic FRET traces after incubation with the target and washing with a wash buffer, this sensing strategy may be used to detect biomarkers even in complex biological matrices that are difficult to assess due to high background. Therefore, these sensors have great potential for multiplexed biomarker detection in the fields of clinical diagnostics and biotechnology.

## Data Availability

Correspondence and requests for materials should be addressed to S.D.

## Supplementary Materials

The following supporting information can be downloaded at: https://www.mdpi.com/article/10.3390/bios13010119/s1, Table S1. DNA sequences for DNA oligonucleotides used in NT sensor assembly; Figure S1. Representative intensity-time and EFRET-time traces showing a highly dynamic behavior for each sensor; Table S2. DNA sequences for single-point mismatch mutants of the target let-7a; Figure S2. Representative intensity-time and EFRET-time traces for each mutant; Figure S3. Validation of sensor specificity.
