# Supplementary material for: Multiplexed smFRET Nucleic Acid Sensing Using DNA Nanotweezers
Source: Biosensors (Basel). 2023 Jan 10;13(1):119. doi: 10.3390/bios13010119 (PMC9856376; doi:10.3390/bios13010119)
Supplement: Supplementary file 1 [file biosensors-13-00119-s001.zip › biosensors-2114393-supplementary.pdf]

biosensors
MDPI

Supporting Information

Multiplexed smFRET Nucleic Acid Sensing Using DNA Nanotweezers

Anisa Kaur †, Roaa Mahmoud †, Anoja Megalathan, Sydney Pettit and Soma Dhakal *

Department of Chemistry, Virginia Commonwealth University, Richmond, VA 23284, USA
* Correspondence: sndhakal@vcu.edu
† These authors contributed equally to this work.

Table S1. DNA sequences for DNA oligonucleotides used in NT sensor assembly.

Name	Sequence (5' → 3')
Cy3 Terminal	TCT TGT GAA CTC CCT ACT ATC CTT AAA CGC ATA TCT CTG A/3Cy3Sp/
Cy3 INT8	TCT TGT GAA CTC CCT ACT ATC CTT AAA CGC AT/iCy3/A TCT CTG A
Cy3 INT12	TCT TGT GAA CTC CCT ACT ATC CTT AAA C/iCy3/GC ATA TCT CTG A
Cy3 INT5	TCT TGT GAA CTC CCT ACT ATC CTT AAA CGC ATA TC/iCy3/T CTG A
Cy5-Trunc.	/5Cy5/GTG TAT GAC CCC TAT ATG TG
Strand 1	ATA GTA GGG AGT TCA CAA GAT GTA TAA GCA AAT ATT TAA A
Bio5'Comp	TTG CAT GCC TGC AGG TCG ACT CTA GTT TTT/Bio-3'/
Splint	AAA CTA GAG TCG ACC TGC AGG CAT GCA ATT TAA ATA TTT GCT TAT ACA
NT-92a	ACAGGCCGGGA-TCAGAGATATGCGTTTAAGG TTTT CACATATAGGGGTCATACAC-CAAGTGCAATA
NT-652	TCACAACCCTA-TCAGAGATATGCGTTTAAGG TTTT CACATATAGGGGTCATACAC-GTGGCGCCATT
NT-107	GATAGCCCTGT-TCAGAGATATGCGTTTAAGG TTTT CACATATAGGGGTCATACAC-ACAATGCTGCT
NT-let7a	AACTATACAAC-TCAGAGATATGCGTTTAAGG TTTT CACATATAGGGGTCATACAC-CTACTACCTCA
DNA92a 22nt	TAT TGC ACT TGT CCC GGC CTG T
DNA652 21nt	AAT GGC GCC ACT AGG GTT GTG
DNA107 23nt	AGC AGC ATT GTA CAG GGC TAT CA
DNA-let7a 22nt	TGA GGT AGT AGG TTG TAT AGT T

Biotin- and fluorophore-modified DNA oligonucleotides were purchased HPLC purified. The four common DNA oligonucleotides found in each NT sensor are highlighted in red. Target binding regions in probe strands are highlighted in blue and magenta. The probe complementary region on fluorophore labeled strands are underlined.

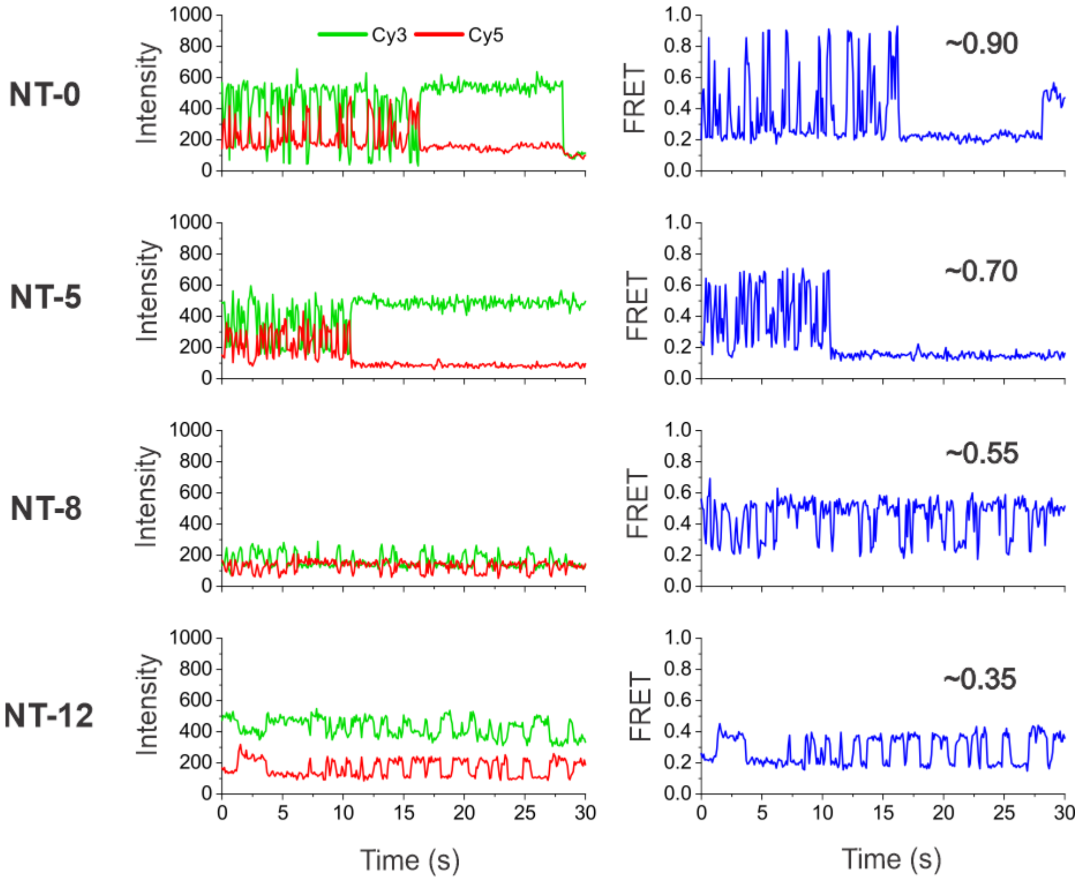

Figure S1. Representative intensity-time and E_{FRET}-time traces showing a highly dynamic behavior for each sensor. Each sensor was characterized individually showing distinct high-FRET states with a mutual low-FRET state of ~0.2 that indicates an unbinding event. Target was added at 100 pM in all experiments.

Table S2. DNA sequences for single-point mismatch mutants of the target let-7a.

Name	Sequence (5' → 3')
Mutant 1 (G11A)	TGA GGT AGT A**A**G TTG TAT AGT T
Mutant 2 (G12A)	TGA GGT AGT AG**A** TTG TAT AGT T
Mutant 3 (T6C)	TGA GG**C** AGT AGG TTG TAT AGT T
Mutant 4 (A17G)	TGA GGT AGT AGG TTG T**G**T AGT T

Mutated bases are highlighted in red. (G11A) implies that the 11th Guanine nucleotide from the 5′-end is altered to adenine.

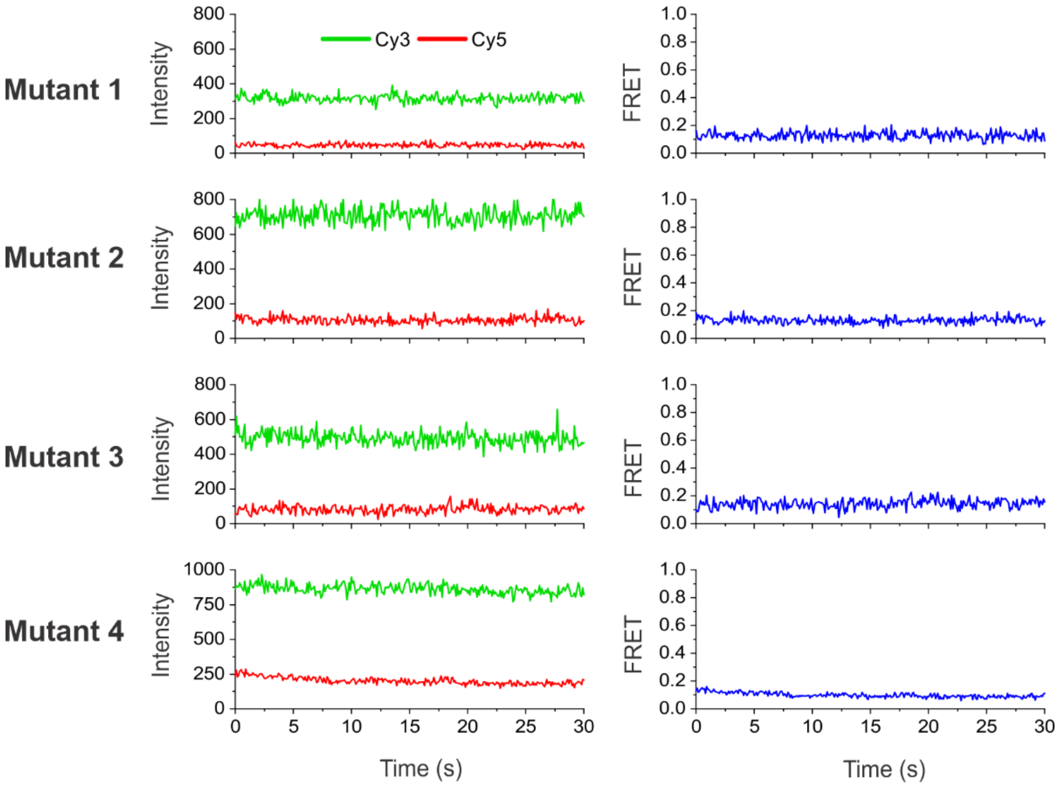

Figure S2. Representative intensity-time and E_{FRET}-time traces for each mutant. A low FRET of ~0.2 was observed for the sets of molecules collected for each mutant, indicating an open conformation of the sensor. Mutants were added at 100 pM in all experiments.

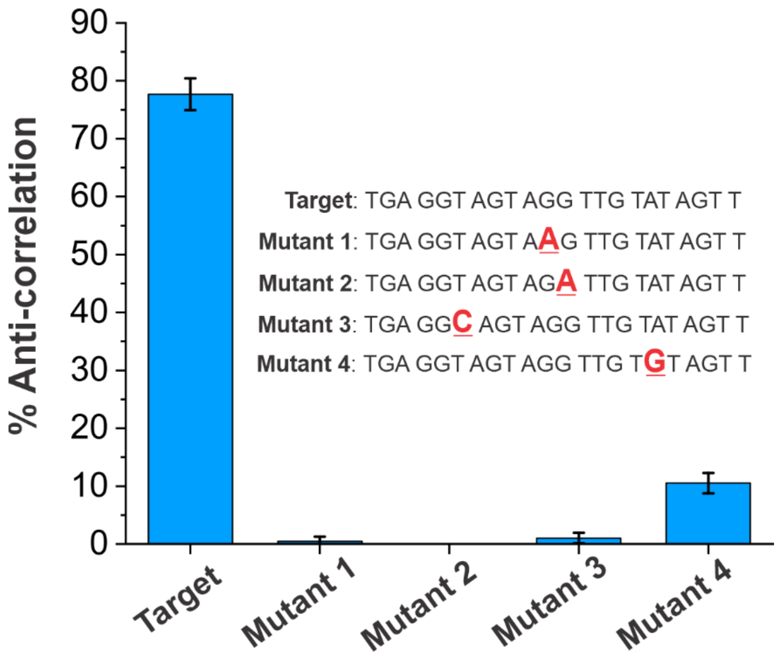

Figure S3. Validation of sensor specificity. Sensor specificity was tested using a nearly saturating concentration of the let-7a target (100 pM) specific to NT-5 and four other mutants. Sequences for the fully complementary target and mutants are shown with the single-point mutations highlighted in red and underlined. All data were obtained from the analysis of 155-213 molecules. Error bars represent standard deviation (SD) calculated after randomly assigning the molecules into three different groups.
